# Characterization of a Crabs Claw Gene in Basal Eudicot Species *Epimedium sagittatum* (Berberidaceae)

**DOI:** 10.3390/ijms14011119

**Published:** 2013-01-08

**Authors:** Wei Sun, Wenjun Huang, Zhineng Li, Haiyan Lv, Hongwen Huang, Ying Wang

**Affiliations:** 1 Key Laboratory of Plant Resources Conservation and Sustainable Utilization, South China Botanical Garden, Chinese Academy of Sciences, Guangzhou 510650, Guangdong, China; E-Mail: djsunwei@gmail.com; 2 University of the Chinese Academy of Sciences, Beijing 100039, China; 3 Key Laboratory of Plant Germplasm Enhancement and Specialty Agriculture, Wuhan Botanical Garden, Chinese Academy of Sciences, Wuhan 430074, Hubei, China; E-Mails: hzauhwj@gmail.com (W.H.); z.li@vu.nl (Z.L.); hyl@wbgcas.cn (H.L.)

**Keywords:** Evo-Devo, nectary development, YABBY, CRC, basal eudicots

## Abstract

The Crabs Claw (CRC) YABBY gene is required for regulating carpel development in angiosperms and has played an important role in nectary evolution during core eudicot speciation. The function or expression of CRC*-*like genes has been explored in two basal eudicots, *Eschscholzia californica* and *Aquilegia formosa*. To further investigate the function of *CRC* orthologous genes related to evolution of carpel and nectary development in basal eudicots, a CRC ortholog, EsCRC*,* was isolated and characterized from *Epimedium sagittatum* (Sieb. and Zucc.) Maxim. A phylogenetic analysis of EsCRC and previously identified CRC*-*like genes placed EsCRC within the basal eudicot lineage. Gene expression results suggest that EsCRC is involved in the development of sepals and carpels, but not nectaries. Phenotypic complementation of the Arabidopsis mutant crc-1 was achieved by constitutive expression of EsCRC. In addition, over-expression of EsCRC in Arabidopsis and tobacco gave rise to abaxially curled leaves. Transgenic results together with the gene expression analysis suggest that EsCRC may maintain a conserved function in carpel development and also play a novel role related to sepal formation. Absence of EsCRC and ElCRC expression in nectaries further indicates that nectary development in non-core eudicots is unrelated to expression of CRC-like genes.

## 1. Introduction

Using integrative approaches, plant evolutionary developmental biology aims to study the molecular basis for the origin of novel traits during plant species evolution and diversification. Elucidation of the functions of important genes in different angiosperm lineages can enhance our understanding of plant evolution. For example, genomic and functional studies based on *Arabidopsis thaliana* and *Antirrhinum majus* have provided knowledge about transcription factors, such as MADS, MYB, bHLH, TCP, NAM/CUC and YABBY, which are responsible for regulating morphological development in angiosperms [1–5].

The YABBY gene family is a small class of transcription factors found exclusively in seed plants, with functions that have been well characterized in Arabidopsis and rice [6–15]. There are six members of the YABBY gene family in Arabidopsis: FILAMENTOUS FLOWER (FIL), CRABS CLAW (CRC), INNER NO OUTER (INO), YABBY2 (YAB2), YAB3 and YAB5 [7,11,16]. The proteins they encode are composed of an *N*-terminal zinc finger domain and a *C*-terminal helix-loop-helix (YABBY domain) [7,15]. Phylogenetic analysis of YABBY genes from extant angiosperm species strongly supports a sister relationship between INO*-*like genes and the common ancestor of CRC-like and FIL-like genes [16]. In Arabidopsis, the “vegetative” YABBYs (FIL, YAB2, YAB3 and YAB5) are expressed in abaxial cells of lateral organ primordia, where they control leaflet initiation and laminar growth [11]. INO expression occurs only in the abaxial domain of the ovule integument [7].

The expression or function of CRC-like genes has been reported for many angiosperms. The abaxial expression of a CRC-like ortholog in carpel walls of two basal angiosperm species, *Amborella trichopoda* and *Cabomba caroliniana*, suggests that an abaxial carpel wall expression pattern was primitively present in the common ancestor of angiosperms [17,18]. In the monocot rice, the CRC ortholog DROOPING LEAF (DL) specifies carpel and floral meristem identity and is also involved in leaf midrib development [9,12,13]. In the basal eudicot *Eschscholzia californica* (California poppy), EcCRC is involved in establishment of carpel polarity and ovule initiation [19]. Abaxial expression in the carpel wall of *Aquilegia formosa*, another basal eudicot, has been detected through *in situ* hybridization, but CRC gene expression has not been observed in the nectary [20]. In core eudicots, the Arabidopsis CRC is the major determinant of carpel growth and fusion and is responsible for floral meristem termination [7,15]. In addition, Arabidopsis CRC has the novel function of regulating nectary identity [7,15]. In other core-eudicot species, such as tobacco and petunia, CRC expression has been detected in both the nectary and the abaxial side of the ovary wall [20]. The carpel development function of CRC orthologs thus appears to be conserved across angiosperms, whereas a role in nectary development has only been observed in core eudicot lineages [20].

The genus *Epimedium* belongs to the Berberidaceae family in Ranunculales, a basal eudicot order. Basal eudicots are a sister group to the core eudicots [19]. *Epimedium* flowers show varying morphology in sepals and petals. Sepals in the outer floral whorl are ovate and not persistent; in contrast, second whorl sepals are notably petaloid and persistent, playing a major role in pollinator attraction. The petals, comprising the third and fourth whorls, are distinguished by a spur and sac. Nectar is made at the tip of the spur [21,22] (Supplementary Figure S1). In the present study, *E. sagittatum* (Sieb. & Zucc.) Maxim was selected to further investigate the function of CRC-like genes in basal eudicots and to examine the hypothesis that CRC exhibits a conserved function in carpels across angiosperms and that the co-option of orthologs in nectary development occurred after the emergence of Ranunculales [20]. CRC orthologs from *Epimedium* were cloned and characterized in a series of experiments that included analysis of gene expression, mutant complementation in Arabidopsis and ectopic expression in Arabidopsis and tobacco.

## 2. Results

### 2.1. Sequence Analyses and Phylogenetic Reconstruction of EsCRC

**Figure 1:**
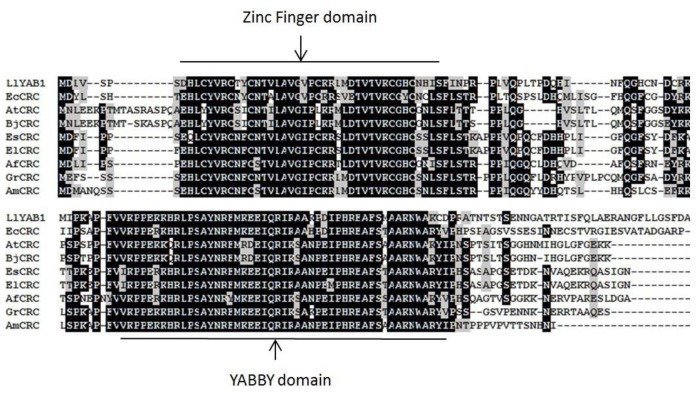
Sequence alignment of Crabs Claw (CRC)-like proteins from Epimedium sagittatum (EsCRC), Epimedium leptorrhizum (ElCRC), Aquilegia formosa (AfCRC), Lilium longiflorum (LlYAB1), Grevillea robusta (GrCRC), Antirrhinum majus (AmCRC), Brassica juncea (BjCRC), Arabidopsis thaliana (AtCRC) and Eschscholzia californica (EcCRC). Conserved domains (Zinc finger domain and YABBY domain) are underlined. Identical residues are highlighted in black and similar residues are highlighted in grey. Detailed information on each protein is located in Supplementary Table S1.

**Figure 2:**
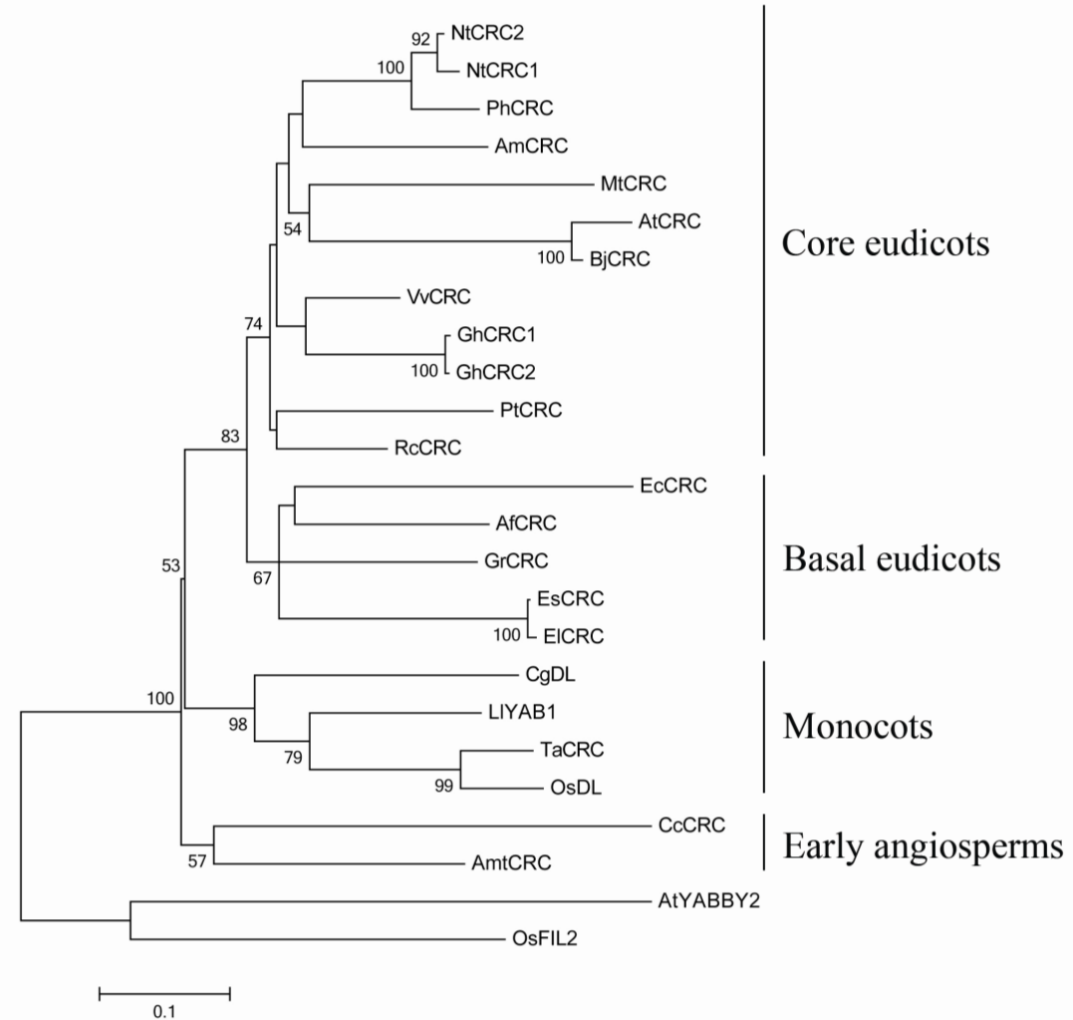
Phylogenetic relationships of CRC-like genes with Arabidopsis YABBY2 (AtYABBY2) and Oryza sativa FIL2 (OsFIL2) as outgroups. Bootstrap values above 50% are indicated. Af, *Aquilegia formosa*; Am, *Antirrhinum majus*; Amt, *Amborella trichopoda*; At, *Arabidopsis thaliana*; Bj, *Brassica juncea*; Cc, *Cabomba caroliniana*; Cg, *Cymbidium goeringii*; Ec, *Eschscholzia californica*; El, *E. leptorrhizum*; Es, *E. sagittatum*; Gr, *Grevillea robusta*; Gh, *Gossypium hirsutum*; Ll*, Lilium longiflorum*; Mt*, Medicago truncatula*; Nt, *Nicotiana tabacum*; Os or*yza sativa*; Ph, *Petunia hybrid;* Pt, *Populus trichocarpa*; Rc, *Ricinus communis*; Ta, *Triticum aestivum*; Vv, *Vitis vinifera*.

The full-length cDNA sequence of EsCRC, including a 534-bp ORF, was isolated from *E. sagittatum*. An orthologous gene, ElCRC, was also cloned from the closely-related *E. leptorrhizum* Stearn using the same primers as in EsCRC. Consistent with other CRC-like proteins from model species, both EsCRC and ElCRC were found to encode proteins possessing a typical zinc finger domain and a YABBY domain (Figure 1). Phylogenetic analysis closely grouped EsCRC and ElCRC with three other CRC-like genes from the basal eudicots *Aquilegia formosa* (AfCRC), *Grevillea robusta* (GrCRC) and *Eschscholzia californica* (EcCRC), indicating that EsCRC and ElCRC are orthologous CRC*-*like genes in the YABBY family (Figure 2).

### 2.2. EsCRC and ElCRC Expression Profiles

**Figure 3:**
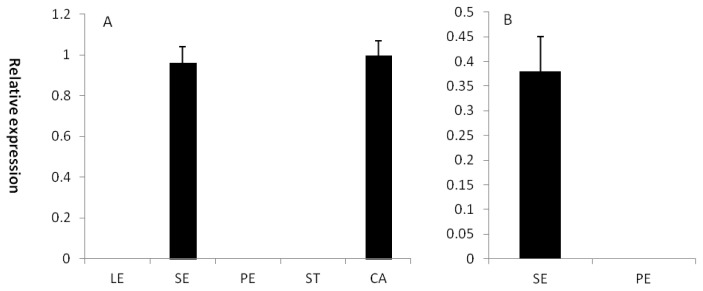
Relative expression of CRC-like genes in (**A**) leaves (LE), sepals (SE), petals (PE), stamens (ST) and carpels (CA) of *Epimedium sagittatum* and (**B**) sepals (SE) and petals (PE) of *Epimedium leptorrhizum*. Error bars represent SE for three technical replicates. The expression of each gene was normalized against the *Epimedium actin* gene.

To investigate the putative role of CRC*-*like genes in basal eudicot *Epimedium* species, gene expression was analyzed in different organs of *E. sagittatum* and *E. leptorrhizum* using quantitative real-time PCR (qPCR). As shown in Figure 3A, EsCRC expression was detected in sepals and carpels, but not in leaves, petals or stamens. To verify the CRC-like gene expression observed in *E. sagittatum* sepals, ElCRC was analyzed in *E. leptorrhizum*: similar expression patterns were observed in sepals, but not in the petals, which possess nectaries at the end of blunt spurs (Figure 3B).

### 2.3. Ectopic Expression of EsCRC Can Rescue Nectary Development in the Arabidopsis crc-1 Mutant

**Figure 4:**
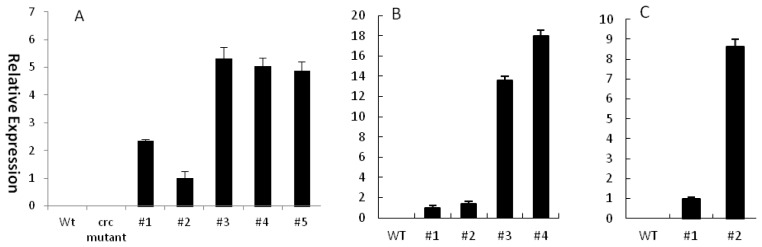
Quantitative real-time PCR analysis of EsCRC transgenes in transgenic lines chosen for further phenotype analysis. (**A**) quantitative real-time PCR (qPCR) results showing EsCRC expression in the Arabidopsis crc-1 mutant transformed with 35S::EsCRC; (**B**) EsCRC expression in transgenic wild-type Arabidopsis; (**C**) qPCR based on relative expression analysis of EsCRC in transgenic tobacco. The expression of each gene was normalized against tubulin in Arabidopsis and actin in tobacco. Error bars represent SE for three replicate reactions.

**Figure 5:**
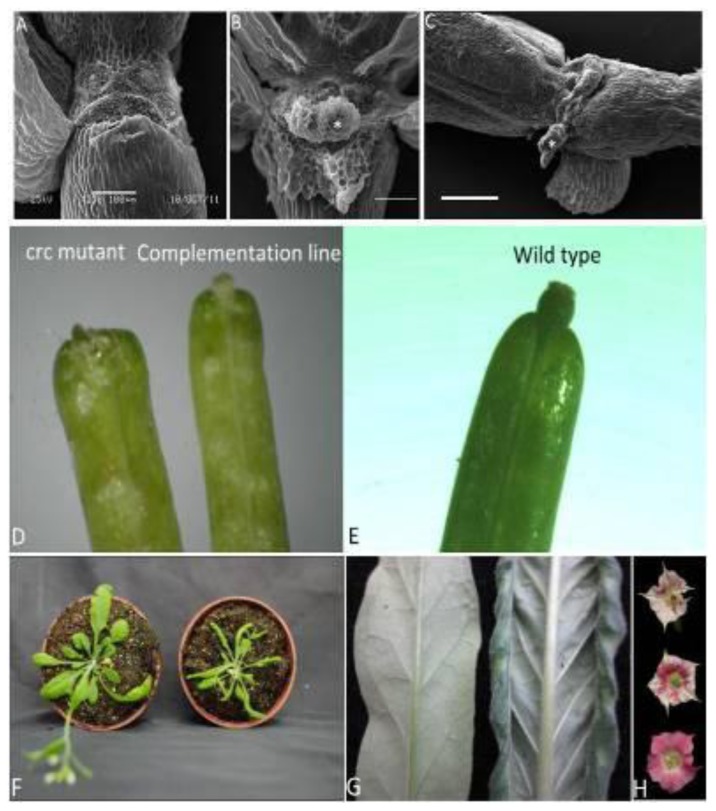
Phenotypic analysis of heterologous constitutive expression of EsCRC in the Arabidopsis crc-1 mutant, wild-type Arabidopsis and tobacco plants. (**A**–**C**) Compared with the Arabidopsis crc-1 mutant (**A**); 35S::EsCRC transgenic plants recovered nectary development (**B**) and rescued carpel defects (**D; right**) relative to nectary morphology (**C**) and fruit morphology in wild-type Arabidopsis (**E**); (**F**) Compared with the wild-type Arabidopsis (**left**), the EsCRC over-expression lines (**right**) showed curling towards the abaxial side; (**G**) Wild-type tobacco leaf (**left**) and curled tobacco leaves (**right**) resulting from constitutive overexpression of EsCRC; (**H**) Petals of the transgenic tobacco were curled (upper) and exhibited loss of anthocyanin (middle and upper) compared with wild-type flowers (bottom). Asterisks indicate the nectary in Arabidopsis. Bars = 100 μM in (**A**) to (**C**).

Constitutive expression of genes from other species is carried out in Arabidopsis mutants to test gene functions [23–26]. To determine whether EsCRC biochemical functions are conserved relative to other CRC-like genes, EsCRC was overexpressed under the control of the cauliflower mosaic virus 35S promoter in the Arabidopsis crc*-*1 mutant. Of 10 independent transgenic plants tested, five lines with EsCRC expression (Figure 4A) showed full complementation of crc-1 carpel and nectary phenotypes and were subjected to further phenotypic characterization (Wild-type [C, E] and mutant crc-1 flowers [A, D] are shown for comparison in Figure 5). The Arabidopsis crc-1 mutant had broader gynoecia than the wild-type, an unfused apex of carpels (Figure 5D) and no nectary development (Figure 5A). An examination of the transgenic plants demonstrated that EsCRC rescued nectary development (Figure 5B) and carpel defects (Figure 5D) in the crc-1 mutant. These results suggest that EsCRC is biochemically equivalent in function to AtCRC. Furthermore, leaves in the transformed complementation lines exhibited abaxial curling as in wild-type Arabidopsis transformed with EsCRC.

### 2.4. EsCRC Influences Abaxial-Adaxial Leaf Formation in Transgenic Arabidopsis and Tobacco

**Figure 6:**
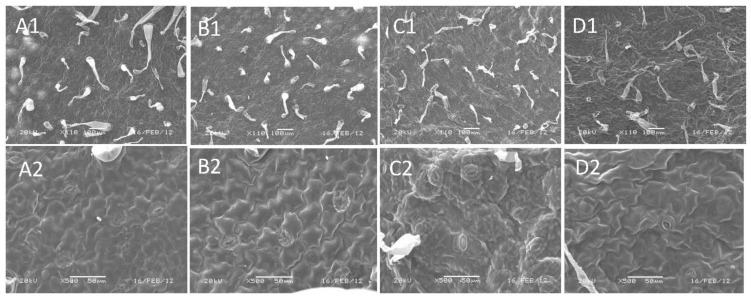
Leaf morphology of wild-type and ectopic overexpression of EsCRC in tobacco. The low magnification pictures provide an overview of (**A1**) abaxial and (**B1**) adaxial surfaces in wild-type tobacco, while high magnification pictures show cell shape, size and type on (**A2**) abaxial and (**B2**) adaxial surfaces of wild-type tobacco. Low and high magnification pictures of tobacco leaves with overexpression of EsCRC show (**C1**, **C2**) abaxial and (**D1**, **D2**) adaxial surfaces with more irregular cell surface and disordered cell arrangement.

It has been demonstrated that CRC orthologs from two monocots are expressed in leaves and can influence leaf development in transgenic Arabidopsis [27,28]. However, we cannot detect EsCRC expression in leaves. To test the possibility that the EsCRC coding region have the capability of affecting the leaf morphology in transgenic plants, we further examined overexpression of EsCRC in transgenic Arabidopsis and tobacco. Out of 10 kanamycin-resistant EsCRC-overexpressing transgenic lines with similar phenotypic changes in each construct, five lines in Arabidopsis and two lines in tobacco (Figure 4B,C) were chosen for detailed analysis. Compared with leaf phenotypes displayed in wild-type Arabidopsis (Figure 5F; left) and tobacco (Figure 5G; left), EsCRC overexpression in tobacco (Figure 5G; right) and Arabidopsis (Figure 5F; right) produced a phenotype with leaves curled towards the abaxial side. When compared with wild-type tobacco (Figure 5H; bottom), EsCRC-overexpressed tobacco exhibited abnormal petals with severely curled edges (Figure 5H; upper) and loss of anthocyanin (Figure 5H; middle and upper). To determine whether the ectopic expression of EsCRC affected epidermal cell morphology of adaxial and abaxial leaves, the abaxial and adaxial surfaces of transgenic and wild-type tobacco plants were examined using a scanning electron microscope. Compared with the abaxial (Figure 6A1,A2) and adaxial epidermis (Figure 6B1,B2) of wild-type tobacco, the transgenic overexpression lines showed more irregular cell surfaces and disordered cell arrangements on both the abaxial and adaxial epidermis (Figure 6C,D). Abaxial epidermis in transgenic lines displayed the most severe abnormalities (Figure 6C).

## 3. Discussion

Functional studies and comparative expression analysis of CRC-like genes in different lineages, including monocots, early-diverging angiosperms, basal eudicots and core eudicots, indicate that CRC-like genes play crucial roles in gynoecium development [7,11,16–19]. For example, a CRC-like gene from the early-diverging angiosperm species *Amborella trichopoda* has been reported to partially complement carpel-related defects in the Arabidopsis crc-1 mutant [27]. In addition, ectopic expression of OsDL, a CRC-like gene from rice, is fully capable of rescuing the severe crc-1 phenotype [27]. Our complementation studies show that EsCRC can rescue carpel defects in the Arabidopsis crc-1 mutant. Collective evidence from heterologous expression of CRC orthologs from *Amborella* or*yza* and *Epimedium* demonstrate that the biochemical functions of CRC are reasonably well conserved. Furthermore, AtCRC, CcCRC, EcCRC and AfCRC expression has been detected in carpel primordia and abaxial walls of carpel primordia [15,17–19]. The expression of EsCRC in carpel tissue observed in our study further supports the notion that CRC-like gene expression in carpels is conserved in angiosperms [18–20]. Heterologous expression of homologous CRC-like genes from rice and *Lilium longiflorum* produce curled leaves in Arabidopsis, and changes in leaf cellular arrangement have been observed in a 35S::LlCRC transgenic Arabidopsis line [27,28]. Our results further confirm the involvement of CRC-like genes in leaf polarization in transgenic Arabidopsis. We were not able to detect EsCRC expression in *Epimedium* leaves, however, indicating that *cis*-regulatory changes may alter the role of EsCRC in *Epimedium* leaves.

In both *E. leptorrhizum* and *E. sagittatum*, transcripts of EsCRC and ElCRC were detected in sepals; this is in contrast to *Eschscholzia californica*, *Aquilegia formosa*, petunia, tobacco and Arabidopsis in which no such expression was observed [7,15,19]. Furthermore, no CRC-like gene expression has been observed in tepals of *L. longiflorum* or *Asparagus asparagoides* [28,29], nor are OsDL genes expressed in rice lemmas or paleae [9]. Taking into consideration the existence of CRC expression in the early-diverging angiosperm *C. caroliniana* [18], we can suggest two explanations for the co-option of CRC-like gene expression in sepals of *Epimedium*. The first explanation assumes that CRC-like gene expression in sepals reflects the ancestral state, implying independent loss of expression in sepals of *Eschscholzia californica*, *Aquilegia formosa* and core eudicots, but not *Epimedium*. The second explanation is that EsCRC expression in sepals arose within *Epimedium*. More investigation of CRC-like gene expression in various species and further functional analysis using gene knock-down techniques, such as virus-induced gene silencing or stable gene transformation, should facilitate understanding of evolution of CRC-like genes.

Obvious nectary defects are observed in Arabidopsis CRC mutants [7] and in tobacco plants in which expression of CRC-like genes has been down-regulated [20]. CRC-like gene expression has been detected in nectaries localized in either extrafloral (*Capparis flexuosa* and *Gossypium hirsutum*) or infloral (petunia and tobacco) organs of representative core eudicots [20]. On the other hand, AfCRC is not transcribed in the tips of spur petals in the basal eudicot *Aquilegia formosa* [20]. In our study, no EsCRC expression was observed in nectary petals, consistent with previous reports that CRC-like genes are not involved in nectary development outside of the core eudicots [19,20]. Moreover, a CRC-like gene from *Amborella trichopoda* has been reported to partially complement carpel-related defects, but not nectary development, in the Arabidopsis crc-1 mutant [17]. However, the ectopic expression of OsDL [17] and EsCRC was able to rescue the severe crc*-*1 phenotype, suggesting that sequence divergence over time in CRC-like genes has somehow affected gene function in rice and *E. sagittatum* compared with *Amborella trichopoda*. Similar functional divergence within AP3-like genes with a paleoAP3 motif derived from gymnosperms and the basal eudicot *Dicentra eximia* resulted in only partial complementation in an experiment involving the Arabidopsis ap3-3 mutant [30,31]. Complementation testing of additional CRC-like gene orthologs from basal angiosperms should provide further insights into evolutionary conservation and divergence of CRC*-*like genes.

## 4. Experimental Section

### 4.1. Study System and Gene Isolation

*Epimedium sagittatum* and *E. leptorrhizum* were grown at the Wuhan Botanical Garden of the Chinese Academy of Sciences and identified by Dr. Yanjun Zhang from the Wuhan Botanical Garden Herbarium. Following extraction from a mixture of inflorescences, leaves and roots using Trizol regeant (Invitrogen, Carlsbad, CA, USA), total RNA was digested with RQ1 RNase-free DNase (Promega, Madison, WI, USA). The EsCRC gene was isolated using a combination of 3′- and 5′-RACE PCR. First-strand cDNA was transcribed from total RNA using Superscript III reverse transcriptase and the poly-T primer 3′-CDS (Invitrogen, Carlsbad, CA, USA). To isolate partial EsCRC, this was followed by 3′ rapid amplification of cDNA ends (3′-RACE) using the 3′-RACE adapter primer SMARTII and degenerate primer ABCRO6 [19]. To obtain the 5′ partial cDNA end of EsCRC, first-strand synthesis was performed using total RNA with SMART II and 5′-CDS primers. Two rounds of 5′ RACE were performed using primer pairs UPM/5′CRCGSP1 for the first-strand PCR and NUP/5′CRCGSP2 for the second-strand PCR. The full-length EsCRC sequence was generated using CRCF and CRCR primers based on the 5′ UTR and 3′ UTR regions. All primers used for gene isolation and confirmation are listed in Supplementary Table S1.

### 4.2. Sequence Acquisition and Phylogenetic Analysis

Sequences of EsCRC, ElCRC and CRC orthologs from various species (Supplementary Table S2) were downloaded from the GenBank database and aligned based on deduced amino acid sequences using online MUSCLE software (EMBL) followed by manual adjustments. Alignments of full length nucleotide sequences were generated using aa2dna software based on the amino acid alignment [22]. Maximum likelihood phylogenetic analyses were performed using Mega 5.0. A general time-reversible (GTR) model with a proportion of invariable sites was selected using Modeltest. Support for each branch was assessed using bootstrap analysis with 1000 bootstrap replicates.

### 4.3. Expression Analysis

To detect the expression of EsCRC and ElCRC, qPCR was performed on RNA from *E. sagittatum* and *E. leptorrhizum* following genomic-eraser purification (Takara, Dalian, China). RNA was obtained from leaves, sepals (outer sepals and inner sepals), petals, stamens and carpels of *E. sagittatum and* sepals and petals of *E. leptorrhizum*. Sepal samples included both outer sepals and inner petaloid sepals. All material was collected during the open flower stage, when nectary was present on petal spur tips. Prior to qPCR, first-strand synthesis and removal of DNA contamination were performed using a PrimeScript RT reagent kit (Takara, Dalian, Japan). qPCR was performed on an ABI7500 Real-Time PCR instrument (Life Technologies, Carlsbad, CA, USA) in 20-μL reaction volumes containing 10 ng cDNA, 10 μM each of primers qCRCF and qCRCR and SYBR Premix Ex Taq II (Takara, Dalian, Japan). The PCR protocol consisted of an initial denaturation stage (95 °C for 30 s, followed by 40 cycles at 94 °C for 3 s and 60 °C for 30 s) and a final dissociation stage (95 °C for 15 s, 60 °C for 1 min and 60 °C for 30 s). The two-delta CT relative quantification method with an *EsActin* internal control [32] was used to normalize expression levels of desired genes in different organs. All experiments were performed with three biological replicates, and three technical replicates were analyzed for each biological replicate. Data were analyzed using ABI7500 software.

### 4.4. Vector Construction and Plant Transformation

To construct the p35 Spro-*EsCRC* vector, full length EsCRC was excised from a PMD19-T vector (Takara, Dalian, Japan) using SalI and KpnI restriction enzymes and then ligated into a pMV binary vector (derived from pBI121) previously digested with XhoI and KpnI. The vectors were then confirmed using sequencing and enzyme digestion. Arabidopsis transformation was carried out using the classic *Agrobacterium*-mediated floral dip method [33]. T_0_ seeds were harvested and screened on half-strength Murashige and Skoog medium supplemented with 50 μg/mL kanamycin, and EsCRC sequences in the resistant seedlings were identified using qPCR. For tobacco transformations, a p35 Spro-EsCRC vector was transferred into the tobacco genome using the *Agrobacterium*-mediated method [34]. Transgenic tobacco plants were screened for further analysis using kanamycin and qPCR. The cDNA of leaf tissue from transgenic plants was used for qPCR.

### 4.5. Scanning Electron Microscopy (SEM) Analysis

Mature leaves from control and transgenic lines were fixed with 3% (*w*/*v*) paraformaldehyde and 0.25% glutaraldehyde in 0.2 N sodium phosphate buffer (pH 7.0) overnight at 4 °C. Fixed samples were rinsed three times with 0.1 M phosphate buffer (pH 7.0). After dehydration in different concentrations of acetone, material was stored overnight in 100% tert-butanol. Samples were dried in a JFD-310 drier (Joel, Tokoyo, Japan) and sputter-coated with carbon using a JFC-1600 anion sputter coating instrument (Joel, Tokoyo, Japan). SEM observations were performed with a JSM-6360LV scanning electron microscope (Joel, Tokoyo, Japan).

## 5. Conclusions

In this study, we isolated a putative CRC-like gene from the basal eudicot species *E. sagittatum*. EsCRC has high identity to other CRC orthologs and contains zinc finger and YABBY domains. As in monocots, basal angiosperms, basal eudicots and core eudicots, EsCRC expression was observed in carpels. In addition, expression was detected in sepals, suggesting a novel function for *EsCRC*. Functional experiments, including overexpression in Arabidopsis and tobacco and complementation of the crc-1 Arabidopsis mutant using p35 Spro-EsCRC demonstrated that EsCRC can influence leaf morphology in Arabidopsis and tobacco and also restore carpel defects and nectary formation in Arabidopsis. No expression was detected in nectary spurs of *E. sagittatum*. These results support a conserved role for CRC in carpel development and suggest that the pathway regulating nectary spur formation in *Epimedium* is different from the pathway controlling nectary development in Arabidopsis.

## Supplementary Information
