# Supplementary material for: Characterization of a Crabs Claw Gene in Basal Eudicot Species Epimedium sagittatum (Berberidaceae)
Source: Int J Mol Sci. 2013 Jan 8;14(1):1119–31. doi: 10.3390/ijms14011119 (PMC3565311; doi:10.3390/ijms14011119)
Supplement: Supplementary file 1 [file ijms-14-01119-s001.pdf]

# Supplementary Information

**Figure S1.** Flowers of *E. sagittatum* (**A**) and *E. leptorrhizum* (**B**). Asterisks indicate the petal with nectariferous tissue.

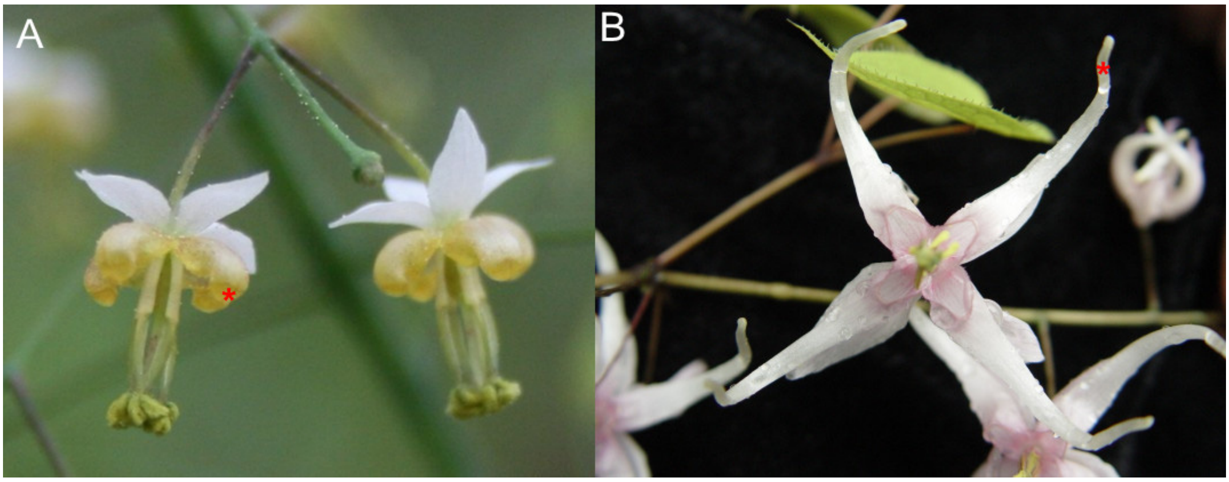


**Table S1.** Primers for cloning and analyzing EsCRC and ElCRC.

| Primers | Sequences (5' to 3') | Usage |
|---|---|---|
| qCRCF (Forward) | CCTGTCCAGCCTCAATGTTT | qRT-PCR |
| qCRCR (Reverse) | CTCGATGGGGTATCTCAGGA | qRT-PCR |
| ABCRC06 | CYGARCATCTYTGCTAYGTCC | 3' RACE |
| 5' CRCGSP1 | ACGATGAAGAATGCGCCTTGAAATCAT | 5' RACE one round |
| 5' CRCGSP2 | GCAGCGGACATAGCAAAGTTGTTCAGA | 5' RACE two round |
| CRCF (Forward) | ATCATCATGGATTTCATCCCACC | Full-length |
| CRCR (Reverse) | ACACCACTCAAGGACTAAGTCC | Full-length |
| 3' CDS | AAGCAGTGGTATCAACGCAGAGTAC(T)30N-1N | RACE |
| SMARTII | AAGCAGTGGTATCAACGCAGAGTACGCGGG | RACE |
| UPML | CTAATACGACTCACTATAGGGCAAGCAGTGGTATCAACGCAGAGT | RACE |
| UPMS | CTAATACGACTCACTATAGGGC | RACE |
| NUP | AAGCAGTGGTATCAACGCAGAGT | RACE |

Es and El indicated *E. sagittatum* and *E. leptorrhizum*, respectively.

**Table S2.** Genes used for phylogenetic analysis and protein alignment.

| Family | Species | Accession No | Name | Type |
|---|---|---|---|---|
| Solanaceae | *Nicotiana tobacum* | AY854799 | NtCRC1 | CRC |
| Solanaceae | *Nicotiana tabacum* | AY854800 | NtCRC2 | CRC |
| Solanaceae | *Petunia x hybrid* | AY854801 | PhCRC | CRC |
| Brassicaceae | *Arabidopsis thaliana* | NM_105585 | AtCRC | CRC |
| Brassicaceae | *Brassica juncea* | XM_002263575 | BjCRC | CRC |
| Malvaceae | *Gossypium hirsutum* | AY854804 | GhCRC1 | CRC |
| Malvaceae | *Gossypium hirsutum* | AY854805 | GhCRC2 | CRC |
| Berberidaceae | *Epimedium sagittatum* | JX560412 | EsCRC | CRC |
| Berberidaceae | *Epimedium leptorrhizum* | JX625141 | ElCRC | CRC |
| Ranunculaceae | *Aquilegia formosa* | EU481798 | AfCRC | CRC |
| Proteaceae | *Grevillea robusta* | Provided by John Bowman | GrCRC | CRC |
| Plantaginaceae | *Antirrhinum majus* | AJ877257 | AmCRC | CRC |
| Liliaceae | *Lilium longiflorum* | EF363135 | LlCRC | CRC |
| Poaceae | *Oryza sativa* | AY494713 | OsDL | CRC |
| Orchidaceae | *Cymbidium goeringii* | HM106986 | CgDL | CRC |
| Papaveraceae | *Eschscholzia californica* | AM946412 | EcCRC | CRC |
| Amborellaceae | *Amborella trichopoda* | AJ877257 | AtCRC | CRC |
| Poaceae | *Triticum aestivum* | AF545436 | TaCRC | CRC |
| Euphorbiaceae | *Ricinus communis* | XM_002512009 | RcCRC | CRC |
| Cabombaceae | *Cabomba caroliniana* | AB553318 | CcCRC | CRC |
| Salicaceae | *Populus trichocarpa* | XM_002316041 | PtCRC | CRC |
| Vitaceae | *Vitis vinifera* | XM_002263575 | VvCRC | CRC |
| Fabaceae | *Medicago truncatula* | XM_003614153 | MtCRC | CRC |
| Poaceae | *Oryza sativa* | NM_001055910 | OsFIL2 | FIL |
| Brassicaceae | *Arabidopsis thaliana* | AF074948 | AtFIL | FIL |

© 2013 by the authors; licensee MDPI, Basel, Switzerland. This article is an open access article distributed under the terms and conditions of the Creative Commons Attribution license (http://creativecommons.org/licenses/by/3.0/).
